# “I’m So Tired”: Fatigue as a Persistent Physical Symptom among Working People Experiencing Exhaustion Disorder

**DOI:** 10.3390/ijerph18168657

**Published:** 2021-08-16

**Authors:** Elín Broddadóttir, Sigrún Ólafsdóttir Flóvenz, Haukur Freyr Gylfason, Þórey Þormar, Hjalti Einarsson, Paul Salkovskis, Jón Friðrik Sigurðsson

**Affiliations:** 1 Department of Psychology, Reykjavik University, 102 Reykjavik, Iceland; sigrunola@ru.is (S.Ó.F.); thoreyt81@gmail.com (Þ.Þ.); jonfsig@ru.is (J.F.S.); 2 Department of Business, Reykjavik University, 102 Reykjavik, Iceland; haukurgy@ru.is; 3 Stett.is, Icelandic Confederation of University Graduates, 105 Reykjavik, Iceland; hjalti@bhm.is; 4 Oxford Centre for Psychological Health, Oxford Institute of Clinical Psychology Training and Oxford Cognitive Therapy Center, Warneford Hospital, Oxford University, Oxford OX3 7JX, UK; paul.salkovskis@hmc.ox.ac.uk; 5 Faculty of Medicine, School of Health Sciences, University of Iceland, 102 Reykjavik, Iceland

**Keywords:** persistent physical symptoms, medically unexplained symptoms, exhaustion disorder, burnout, depression, anxiety, functional impairment, stress-related disorder

## Abstract

Fatigue is widespread in the population, particularly among working people. Exhaustion disorder (ED), a clinical manifestation of burnout, is common, but, after treatment, about one-third still experience fatigue and other physical symptoms. We propose that in some instances, fatigue as a persistent physical symptom (PPS) might be a more appropriate formulation of ED patients’ fatigue problems, and we suggest that ED patients who meet fatigue PPS criteria will differ from other ED patients in terms of psychological distress, non-fatigue PPSs and functional impairment. Questionnaires were sent to 10,956 members of a trade union of which 2479 (22.6%) responded. Of 1090 participants who met criteria for ED, 106 (9.7%) met criteria for fatigue as a PPS. Participants who met fatigue PPS criteria scored on average higher on measures of depression, anxiety and functional impairment and were more likely to have clinically significant scores. Moreover, they had 27 times higher odds of meeting other PPS subtypes and reported more non-fatigue PPS subtypes, suggesting a more complex health problem. Specific evidence-based interventions are available for both ED and PPSs, and therefore, it is crucial to accurately formulate the fatigue problem reported by patients to provide appropriate treatment.

## 1. Introduction

Fatigue is widespread in the general population [1,2,3], frequently presented in primary care [4,5,6] and increasingly reported by the working population [7,8,9]. Fatigue in the workforce has attracted renewed attention, as it likely influences workers’ motivation, performance, sick leave and disability [10] in addition to general mortality [11] with accompanying personal, organisational and societal costs [12,13]. The understanding of the pathophysiology and underlying mechanisms of fatigue is limited, but fatigue symptoms may indicate a wide variety of physical, neurological and psychological conditions [14]. For example, fatigue is a core symptom of several well-defined, unexplained conditions, common among working people, for which specific evidence-based interventions have been developed, including exhaustion disorder (ED), a clinical manifestation of burnout [15].

ED is a stress-related condition with fatigue as a central symptom, accompanied by other exhaustion-related symptoms. ED’s diagnostic criteria were proposed by the Swedish National Board of Health and Welfare in 2003 as a clinical operationalisation of burnout [15,16,17]. The prevalence of ED in the working population ranges from 10 to 23% [10,18,19,20,21,22,23], and ED is associated with reduced work ability and sickness absences [24]. Individuals fulfil the diagnostic criteria for ED if they have experienced physical and mental symptoms of fatigue for at least 2 weeks and if the symptoms can be attributed to an identifiable stressor that has been present for at least 6 months. People must experience markedly reduced mental energy and at least four other symptoms of exhaustion, such as problems with concentration, sleep disturbances, irritability or physical symptoms [24].

ED and burnout interventions have both focused on the individual experiencing stress as well as the workplace environment and its stressors [25,26]. While evidence is inconclusive on workplace interventions, Cognitive Behavioural Therapy (CBT) and multimodal treatment for stress-related disorders, including ED, show promising results [26,27,28,29]. CBT and multimodal treatment seem to be effective in reducing psychological and stress-related physical symptoms but less effective when it comes to the number of sick leave days and returning to work [28,30,31,32,33]. Between 60 and 80% of ED patients do not meet criteria post-treatment [31,34,35], and between 70 and 80% of ED patients return to work within a year regardless of intervention [36]. Despite these promising results, about one-third of ED patients continue to have disabling fatigue after being treated [31,35,37,38] and about 20% continue to have multiple physical symptoms in addition to their fatigue [32]. This implies that around 5% of the workforce is still experiencing disabling fatigue after treatment. In light of this, we aim to explore the possibility that an alternative formulation of fatigue problems experienced by ED patients might in some instances be more appropriate, namely as a persistent physical symptom (PPS).

“PPS” is a new umbrella term used for physical symptoms that are persistent, distressing and have no known underlying biological cause. Many different terms have been used to describe such unexplained symptoms, including “medically unexplained symptoms” and “functional somatic symptoms”. The latest versions of the *Diagnostic and Statistical Manual of Mental disorders* (*DSM-5*) [39] and the *International Classification of Diseases* (*ICD-11*) [40] contain updated conceptualisations of somatic symptom disorder (*DSM-5*) and bodily distress disorder (*ICD-11*), which, although compatible with the definition of PPSs, no longer require the symptoms to be medically unexplained, and they can therefore be diagnosed alongside explained conditions. We use the term “PPS” as it encompasses strictly unexplained physical symptoms and is generally preferred by patients [41,42]. Several medically unexplained conditions with fatigue as the central or accompanying symptom fall under the umbrella term of PPSs, such as chronic fatigue syndrome, fibromyalgia, irritable bowel syndrome and non-cardiac chest pain. Some argue that PPSs are better defined as one condition rather than many, as it is common to meet criteria for multiple unexplained conditions, and there is considerable overlap in case definitions as well as non-symptom associations [43,44,45,46,47]. PPSs are divided into seven subtypes, which are, in addition to persistent fatigue, sleep difficulties, chronic pain, gastrointestinal problems, chest and heart conditions and gynaecological problems. In order to meet criteria for any PPS subtype, including persistent fatigue, the symptom must not be explained by any verifiable condition, disease or the influence of drugs or substances; the symptom must have been present for 6 months; and the symptom must significantly influence people’s daily functioning. 

PPSs are common in all health care settings [48,49,50] and are associated with high rates of disability [51,52,53], comorbid psychological disorders [54,55,56,57,58], unemployment [59,60] and work disability [55,58,61,62]. Additionally, PPS patients have a poor prognosis, especially when multiple symptoms are present [63], but studies show that CBT is an effective intervention for PPSs [64,65,66,67] and in particular persistent fatigue [68,69,70]. 

Little is known about the overlap between ED and PPSs. What we know comes from studies comparing burnout and chronic fatigue syndrome. Such comparison studies conclude that burnout and chronic fatigue syndrome overlap considerably but are nonetheless separate conditions [34,71,72,73]. Studies have found that burnout and chronic fatigue syndrome patients differ in terms of general health and perceived general health [72], causal attributions [71,72], emotional awareness capacity [74], health-related quality of life [73] and work absenteeism [34]. Further, people suffering from burnout have higher rates of recovery than patients with chronic fatigue syndrome [34].

In this study, we aimed to identify ED patients whose fatigue meets PPS criteria and explore whether they differ from other ED patients in terms of psychological distress, non-fatigue PPSs and functional impairment, inspecting whether this alternative formulation of the fatigue problem might be more appropriate. PPSs are associated with poor prognosis and complex psychophysiological problems, and we thus presume that the ED participants whose fatigue meets PPS criteria will be more depressed, anxious and functionally impaired and more likely to meet criteria for other PPS subtypes than participants whose fatigue does not meet PPS criteria. 

## 2. Materials and Methods

### 2.1. Participants and Procedures

Participants were recruited from 13,000 members of the Icelandic Confederation of University Graduates (BHM), an umbrella organisation of 27 trade unions from public and private sectors. A survey was sent via email to 10,956 members from the BHM’s main office email account with two reminders a week apart. Of the 2479 (22.6%) who initially responded, 1898 (76.6%) were women and 581 (23.4%) men. However, in line with the aims of the study, the final sample consisted of 1090 (44%) participants who met criteria for ED. Demographic characteristics of the participants are shown in Table 1. The study was approved by The National Bioethics Committee of Iceland (no. 18-044).

### 2.2. Measures

Demographic information. The survey included demographic questions on gender, age, marital status, education and employment (Table 1). 

Self-rated Exhaustion Disorder (s-ED). The s-ED was used to assess whether participants met criteria for ED [24]. The instrument is a self-report checklist consisting of four items based on the diagnostic criteria for ED. Respondents are asked whether they have felt physically and/or mentally exhausted in the last two weeks; whether they believe the exhaustion to be caused by stress exposure over the last 6 months or more; and whether they have experienced any of the following symptoms in the last two weeks: concentration or memory problems, markedly reduced capacity to tolerate demands or to work under pressure, emotional instability or irritability, sleeping problems, physical weakness or being more easily fatigued and physical symptoms (such as muscular pain, chest pain, palpitations, gastrointestinal problems, vertigo or increased sensitivity to sound). Finally, respondents are asked whether these complaints have markedly decreased well-being and/or functional capacity. The answer options are ‘Yes, to a great extent’, ‘Yes, somewhat’ and ‘No, not at all’. The criteria for ED are met if respondents experience exhaustion, can identify a stressor, experience at least four related symptoms and are functionally impaired. The s-ED has shown good construct validity [17,22,24,75]. 

Persistent Physical Symptom Checklist (PPSC). PPSC was used to assess whether participants met criteria for persistent fatigue and other subtypes of PPSs [6]. It is a self-report checklist used to identify people experiencing problems with seven subtypes of unexplained physical symptoms that interfere with functional capacity, i.e., sleep problems, pain, fatigue and muscle problems, gastrointestinal problems, problems with the heart and chest, dizziness or related problems and gynaecological problems. For each type of physical symptom, respondents are asked four questions: whether they have experienced the symptom for more than six months (except one month for sleep problems), whether their symptoms have a known cause and, if it is known, what it is. Finally, respondents are asked to indicate on a 9-point scale (0 = Not at all, 8 = Very severely) to what extent each problem interferes with their lives. The criteria for a particular PPS type are met if the problem has been present for more than six months, its cause is not known or the cause is an unexplained syndrome and the problem interferes with daily life, which is 4 or higher on the 9-point interference scale. Persistent fatigue as a PPS was assessed by asking whether respondents had experienced “excessive fatigue that is not alleviated by rest”. 

The Patient Health Questionnaire-9 (PHQ-9): Depressive symptoms were measured with PHQ-9 [76], a 9-item self-report instrument that measures severity of depression over the last two weeks (Cronbach’s *α* = 0.80). Items are rated on a 4-point scale from 0 (not at all) to 3 (nearly every day) with total scores ranging from 0–27 points. A total score of 10 or more reliably differentiates between those who meet criteria for depression and those that do not [77]. 

The Generalized Anxiety Disorder-*7* *(GAD-7):* The participants also completed GAD-7 (Cronbach’s *α* = 0.88). GAD-7 is a 7-item self-report instrument that measures symptoms of general anxiety over the last two weeks [78]. Items are rated on a 4-point scale from 0 (never) to 3 (nearly every day), and total scores range from 0‒21 points. A total score of 10 or more reliably differentiates between those who meet criteria for general anxiety disorder and those that do not [78]. 

The Work and Social Adjustment Scale (WSAS): Impaired functioning was assessed with the WSAS (Cronbach’s *α* = 0.87) [79]. WSAS measures impaired functioning in five areas of everyday life: the ability to work, manage a home, form and maintain close relationships and engage in social and leisure activities. The scale consists of five items measured on a 9-point scale (0 = Not at all, 8 = Very severely) with a total score that ranges from 0 to 40. Scores between 10 and 20 have been associated with significant functional impairment and scores over 20 a more severe pathology [79]. 

### 2.3. Statistical Analysis

Statistical analysis was carried out with SPSS 26 (IBM, Armonk, NY, USA). First, chi-square (χ^2^) tests were used to determine whether the demographic characteristics of participants differed depending on meeting fatigue PPS criteria. Second, *t*-tests and chi-square tests were used to determine whether mean and clinically significant scores on PHQ-9, GAD-7 and WSAS differed between participants whose fatigue met PPS criteria and those that did not. Third, chi-square tests were used to estimate the odds of meeting criteria for any non-fatigue PPS subtypes between participants whose fatigue met PPS criteria and those that did not. Consequently, a binary logistic regression analysis estimated whether the number of reported non-fatigue PPSs predicted whether participants’ fatigue met PPS criteria while controlling for symptoms of depression and general anxiety. 

## 3. Results

### 3.1. Demographic Characteristics

Of the 1090 participants, who all met criteria for ED, 106 (9.7%) experienced fatigue so persistent that it met PPS criteria (Table 1). Comparisons revealed that participants whose fatigue met PPS criteria were more likely to be women than participants whose fatigue did not meet PPS criteria. The groups did not significantly differ on other demographic variables (*p* > 0.05).

### 3.2. Psychological Distress

As can be seen in Table 2, participants whose fatigue met PPS criteria reported on average more symptoms of depression, anxiety and functional impairment than participants whose fatigue did not meet PPS criteria. There was a significant association between having fatigue that met PPS criteria and having symptoms of depression (PHQ-9), general anxiety (GAD-7) and functional impairment (WSAS) in the clinical range. Participants whose fatigue met PPS criteria were around two times more likely to have depression and anxiety symptoms in the clinical range and nine times more likely to have clinically significant functional impairment. 

### 3.3. Number of Non-Fatigue PPSs

**Figure 1:**
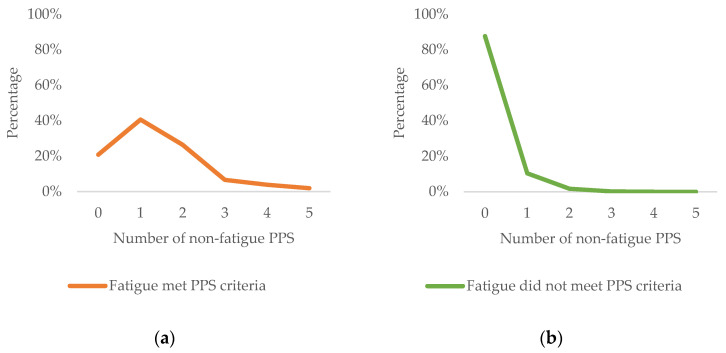
(**a**) Percentage of participants whose fatigue met PPS criteria and who met criteria for none, one or more non-fatigue PPS. (**b**) Percentage of participants whose fatigue did not meet PPS criteria and who met criteria for none, one or more non-fatigue PPS.

Participants with fatigue as a PPS had 27 times higher odds (95% CI = 16.4−45.2) of meeting criteria for any other PPS subtypes (79%) compared to participants whose fatigue did not meet PPS criteria (12%), χ^2^(1) = 280.88, *p* < 0.001. Moreover, participants whose fatigue did not meet PPS criteria generally did not meet criteria for any PPS subtype (88%), and the prevalence declines as the number of PPSs increase (Figure 1). Around 10% met criteria for one other subtype of PPSs, most commonly sleep problems and pain. However, among participants whose fatigue met PPS criteria, it was most common to meet criteria for one (40%) or two (26%) non-fatigue PPS subtypes, while 21% met criteria for fatigue as a PPS only. Pain and sleep problems are also the most common non-fatigue PPS criteria met among people whose fatigue met PPS criteria. A logistic regression analysis suggested that the number of reported non-fatigue PPSs significantly predicted whether fatigue PPS criteria were met while controlling for symptoms of depression and anxiety (data not shown). 

## 4. Discussion

Exhaustion disorder and persistent fatigue as a PPS have very similar symptomologies, as both have fatigue as the core symptom and are associated with sickness absence and work disability [24,80]. Despite this overlap, they are separate disorders with a differing prognosis [34,72] and specialised interventions. We proposed that a portion of people who met ED criteria were experiencing fatigue that met criteria for PPS and that they would differ from other participants by experiencing more psychological distress and multiple PPSs. The results confirmed our conjecture, as participants whose fatigue met PPS criteria were more likely to have clinical levels of depression, anxiety and functional impairment, and a substantial proportion met criteria for multiple PPS subtypes.

Participants whose fatigue met PPS criteria scored on average higher on measures of depression, anxiety and functional impairment than their counterparts whose fatigue did not meet PPS criteria. Yet, the effect sizes were moderate. When using the measures’ clinical cut-points, the results showed that participants whose fatigue met PPS criteria were twice as likely to have clinical levels of depressive and anxiety symptoms than participants whose fatigue did not meet PPS criteria and nine times more likely to have clinically significant functional impairment. Our results align with previous studies that indicate that 40–60% of PPS patients meet criteria for concurrent depressive and anxiety disorders [6,52,56,81], but in our sample, 63% and 46% of participants with fatigue as a PPS had clinical levels of depression and general anxiety, respectively. 

Participants whose fatigue met PPS criteria had 27 times higher odds of meeting any other PPS subtypes compared to participants without fatigue as a PPS, with 80% having two or more subtypes of PPSs, including persistent fatigue. Furthermore, depressive and anxiety symptoms did not predict whether participants’ fatigue met PPS criteria, while the number of non-fatigue PPSs did. This corroborates the clinical importance of multiple PPSs. Previous research has shown that people with PPSs are a vulnerable group with poor functioning and a high chance of comorbidity with other PPSs and psychological disorders [52,55,82,83], which suggests that the ED patients that meet criteria for fatigue as a PPS are likely suffering from a more complex problem and require different treatment. 

We highlighted the very similar symptomologies of ED and persistent fatigue as a PPS. Fatigue that is not alleviated by rest is the primary symptom in both conditions and is often accompanied by sleep problems, concentration problems, pain and gastrointestinal problems among other symptoms [6,15]. The clearest differences in the diagnostic criteria are as follows: Firstly, symptoms in ED are stress related and are developed in response to an identifiable stressor. Although the literature indicates that post-traumatic stress and everyday stress may be precipitating, predisposing or perpetuating factors when it comes to PPSs [84] and fatigue-centred unexplained syndromes, such as chronic fatigue syndrome [73] and fibromyalgia [85,86,87], this association is contested and is not a requisite. Secondly, the timeframes of ED and fatigue as a PPS differ and might be a clinically important element when formulating the problem experienced by the patient and, hence, when providing treatment. Although ED has a long preface of stress, fatigue need only have been present for two weeks, and the onset is often sudden and intense [88]. On the other hand, fatigue as a PPS must be present for at least 6 months and is therefore long lasting, has a more chronic course and higher rates of reoccurrence [34]. Relatively little is known about the clinical course of ED [34,88], but studies suggest that symptom duration before intervention is highly important to recovery from ED [31] and fatigue in general [89]. 

There are several limitations to this study. Although the sample was large and consisted of working people only, it was non-random with self-selected participants. Additionally, the sample consisted of university educated employees only, and women were overrepresented. Further, our results are based on self-reporting of ED symptoms and PPSs; thus, it would be desirable to confirm our results with more robust methods, e.g., clinical interviews with standardised instruments and diagnostic evaluation of health care professionals. Furthermore, it would be of value to explore the association between ED, PPSs and diagnosed mental disorders to more effectively isolate associations with psychological distress and functional impairment by controlling for diagnosis and current treatment regimens.

Formulating the fatigue problem reported by patients accurately is crucial from a public health perspective as well as for providing appropriate treatment. Although the treatment interventions for ED and persistent fatigue as a PPS share some similarities, there are crucial differences. ED multimodal treatment is focused on stress reduction, which includes increasing physical activity and psychoeducation on lifestyle topics, insomnia and mental health CBT [31]. CBT for persistent fatigue as a PPS places focus on understanding the interrelation between mental and physical symptoms, psychoeducation and creating routines with activity pacing [90]. Our results suggest that among ED patients are people experiencing PPSs who might benefit from more specific PPS treatment. Previous studies indicate that at an 18 month follow-up after multimodal treatment, 33% still meet criteria for ED [31] and 20% still experience six or more physical symptoms in addition to their fatigue [32]. In our sample, 10% of ED participants met criteria for fatigue as a PPS and 80% of those reported multiple PPSs. We thus speculate that for a substantial subgroup of ED patients, PPSs might be a more accurate formulation of their problems, as they are experiencing a more complex and persistent form of fatigue mixed with other physical symptoms that need specific intervention. 

## 5. Conclusions

ED and fatigue as a PPS both have fatigue as a central symptom, both are often accompanied by other physical symptoms and both influence disability and work absenteeism [24,55,61]. Yet, they have a differing clinical course, prognosis and treatment [34,88,90]. Our results suggest that among ED patients, there might be a group of patients experiencing a more complex problem of multiple PPSs that warrants a specialised approach to treatment. With increasing societal and personal costs due to fatigue among the working population, it is crucial that clinicians and researchers continue to broaden the understanding and research of distinctive fatigue conditions, develop tools and resources to differentiate accurately between fatigue conditions and advance appropriate evidence-based treatment options. 

## Figures and Tables

**Table 1:** Demographic characteristics of participants whose fatigue met or did not meet persistent physical symptom (PPS) criteria.

Demographic Variables	All Participants	Fatigue Met PPS Criteria	Fatigue Did Not Meet PPS Criteria	χ^2^
Gender				
Female	892 (81.8%)	100 (94.3%)	792 (80.5%)	12.351 ***
Male	198 (18.2%)	6 (5.7%)	192 (19.5%)	
Age				
30 years or younger	60 (5.5%)	6 (5.7%)	54 (5.5%)	0.006
31–50 years	699 (64.1%)	68 (64.2%)	631 (64.1%)	
51–70 years	331 (30.4%)	32 (30.2%)	299 (30.4%)	
Education completed				
Undergraduate degree	465 (42.7%)	52 (49.1%)	413 (42.0%)	1.964
Graduate degree	625 (57.3%)	54 (50.9%)	571 (58.0%)	
Marital status				
Single	128 (11.8%)	17 (16.2%)	111 (11.4%)	2.971
Married/relationship	880 (81.4%)	79 (75.2%)	801 (82.1%)	
Separated/widowed	73 (6.8%)	9 (8.6%)	564 (6.6%)	
Employment				
Executives and managers	290 (26.9%)	32 (30.8%)	258 (26.5%)	3.292
Specialists	398 (36.9%)	32 (30.8%)	366 (37.6%)	
Specialists in human services	335 (31.1%)	36 (34.6%)	299 (30.7%)	
Specialised workers	9 (0.8%)	0 (0.0%)	9 (0.9%)	
Retail, service and other	46 (4.3%)	4 (3.8%)	42 (4.3%)	
Total	1090 (100%)	106 (9.7%)	984 (90.3%)	

Note: *** *p* < 0.001.

**Table 2:** Comparison of depressive symptoms, anxiety symptoms and functional impairment between participants whose fatigue met or did not meet PPS criteria.

Measures	All Participants	Fatigue Met PPS Criteria	Fatigue Did Not Meet PPS Criteria	Sig.	Effect Size
Means	M (SD)	M (SD)	M (SD)	*T*	*r*
PHQ-9	9.34 (4.6)	11.63 (5.0)	9.08 (4.5)	−5.36 ***	0.17
GAD-7	7.73 (4.6)	9.49 (5.1)	7.52 (4.5)	−4.14 ***	0.13
WSAS	13.04 (8.4)	20.07 (8.6)	12.22 (8.0)	−9.33 ***	0.28
Cut-offs	*N* (%)	*N* (%)	*N* (%)	χ^2^	OR [95% CI]
PHQ-9_≥10_	389 (35.7%)	60 (56.6%)	392 (33.4%)	16.44 ***	2.43 [1.6–3.8]
GAD-7_≥10_	290 (26.6%)	44 (41.5%)	246 (25.0%)	10.98 ***	2.04 [1.3–3.1]
WSAS_≥10_	581 (53.3%)	91 (85.8%)	490 (49.8%)	44.37 ***	9.29 [4.3–20.3]

Note: *** *p* < 0.001 M = mean. SD = standard deviation. OR = odds ratio. CI = confidence interval.

## Data Availability

The data presented in this study are available on request from the corresponding author. The data are not publicly available due to ethical restrictions.
